# Gold Nanoparticles Grafted with PLL-*b*-PNIPAM: Interplay on Thermal/pH Dual-Response and Optical Properties

**DOI:** 10.3390/molecules23040921

**Published:** 2018-04-16

**Authors:** Hui-Juan Li, Peng-Yun Li, Li-Ying Li, Abdul Haleem, Wei-Dong He

**Affiliations:** 1 CAS Key Laboratory of Soft Matter Chemistry, Department of Polymer Science and Engineering, University of Science and Technology of China, Hefei 230026, China; lhj2015@mail.ustc.edu.cn (H.-J.L.); pyli@mail.ustc.edu.cn (P.-Y.L.); liliyingscu@163.com (L.-Y.L.); haleem@mail.ustc.edu.cn (A.H.); 2 Fourth Technique Division, Third Institute of China Aerospace Science and Industry Corp, Beijing 100048, China

**Keywords:** gold nanoparticles, PNIPAM, poly(l-lysine), thermal response, pH response, surface plasmon resonance, peptide conformation

## Abstract

Narrowly distributed poly(l-lysine-*b*-*N*-isopropylacrylamide) (PLL-*b*-PNIPAM) was prepared through ring-opening polymerization of ε-benzyloxycarbonyl-l-lysine *N*-carboxy-α-amino anhydride and atom transfer radical polymerization of NIPAM, followed with the removal of ε-benzyloxycarbonyl group. Then gold nanoparticles (AuNPs) grafted with PLL-*b*-PNIPAM (PNIPAM-PLL-AuNPs) were obtained by the reduction of chloroauric acid with sodium citrate in the presence of PLL-*b*-PNIPAM. PNIPAM-PLL-AuNPs and its precursors were thoroughly characterized by proton magnetic resonance spectroscope, Fourier transform infrared spectroscope, UV-vis spectroscope, transmission electron microscopy, dynamic light scattering, thermogravimetric analysis, and circular dichroism. The obtained PNIPAM-PLL-AuNPs exhibited high colloid stability even at strong alkaline (pH = 12) and acidic (pH = 2) conditions. The thermal and pH dual-responsive behaviors of the grafting PLL-*b*-PNIPAM chains was observed to be affected by AuNPs, while not for the secondary structure of PLL chains. Correspondingly, the surface plasmon resonance (SPR) of AuNPs was found to be sensitive to both pH value and temperature. A blue shift in the SPR happened both with increasing pH value and increasing temperature. The stimuli-response was reversible in heating-cooling cycles. The gold nanoparticles with both pH and temperature response may have potential applications in biomedical areas and biosensors.

## 1. Introduction

Metal nanoparticles, especially gold nanoparticles (AuNPs), have attracted a great deal of attention in recent decades owing to their unique physical and chemical properties [1,2,3,4], and their potential applications in optical and microelectronic devices, biomedicine, as well as in catalysis [5,6,7,8,9,10]. Generally, AuNPs are protected by an organic layer composted of small surfactants [11,12,13] and polymers [14,15]. 

Recently, nanoparticles capped with functional polymers have been extensively studied [16,17]. These polymers are important for stabilizing AuNPs against aggregation and maintaining AuNPs’ properties. The polymer-protected AuNPs can also obtained as stable concentrated dispersions or re-dispersible powders [18,19,20], and behave as robust molecules that are amenable to characterization by a variety of analytical methods [21,22]. However, among the polymer-protected AuNPs, most of polymer matrixes only act as a support for nanoparticles. AuNPs stabilized by stimuli-responsive polymers are highly desirable in some specific areas such as nano-medicine, nano-sensor, bioimaging. Up to now, reports of stable gold nanoparticles with pH and thermo-sensitive polymers have been increasing [23,24,25,26]. Considering the localized surface plasmon resonance (LSPR) of AuNPs coated with the responsive polymeric shell, LSPR spectral shift will happen since the surrounding environment changes. By adjusting the stimulation, the wavelength and relative scattering/absorption properties of gold nanoparticle could be altered. Due to this, polymer-protected AuNPs can be the outstanding candidate for analyst, sensor, and spectroscopy applications [27,28,29].

Poly(l-lysine) (PLL) is an excellent, yet probably the simplest model peptide which undergoes a remarkable pH-induced conformational transition. At acidic or neutral conditions, PLL chains adopt random coil conformation, while adopting α−helix conformation owing to the deprotonation of lysine residues of PLL under alkaline conditions [30,31,32]. Lin and coworkers [33] found that owning to the coil-to-helix transitions of PLL and PLGA, the aggregation of a linear-dendron-like polyampholyte based on positively charged PLL and negatively charged poly(l-glutamic acid) (PLGA) shows pH responsiveness. As reported by Liu et al. [25], the prepared PMEO_2_MA-*b*-PDMAEMA-AuNPs and PDMAEMA-*b*-PMEO_2_MA-AuNPs, where in the former, the pH- and thermo-responsive poly[*N*,*N*′-(dimethylamino)ethyl methacrylate] (PDMAEMA) acts as the inner block and the thermo-responsive poly[2-(2-methoxyethoxy)ethyl methacrylate] (PMEO_2_MA) serves as the outer block; in the latter, the situation is the opposite. Interestingly, a significant difference was found that only the former exhibited thermo-induced aggregation in salty solutions. Schatz found that after deprotonated carboxylate groups of carboxylate groups from 11-mercaptoundecanoic acid (11-MUA), the LSPR of Ag nanoparticles modified with polypeptide poly-l-lysine (PL) and 11-MUA suffered red shifts of 5 nm [34]. 

To illustrate the performance of multi-responsive polymer-protected nanoparticle, herein, we prepared PNIPAM-*b*-PLL-AuNPs, where the block copolymer was bound to the gold surface by the inner pH-responsive PLL block, and the thermo-sensitive PNIPAM serves as the outer block. The properties of the gold nanoparticles protected with stimuli-responsive diblock copolymers can be modified by varying pH and temperature of the AuNPs aqueous dispersion. The results indicate both stimuli were shown to affect the optical properties of the particle. Interestingly, these gold nanoparticles protected by inner PLL block and outer PNIPAM block exhibited good stability at room temperature even at strong alkaline conditions (pH = 12), due to the effective steric stabilization brought by the highly hydrated long PNIPAM chains on the gold surface.

## 2. Results and Discussion

**Scheme 1:**
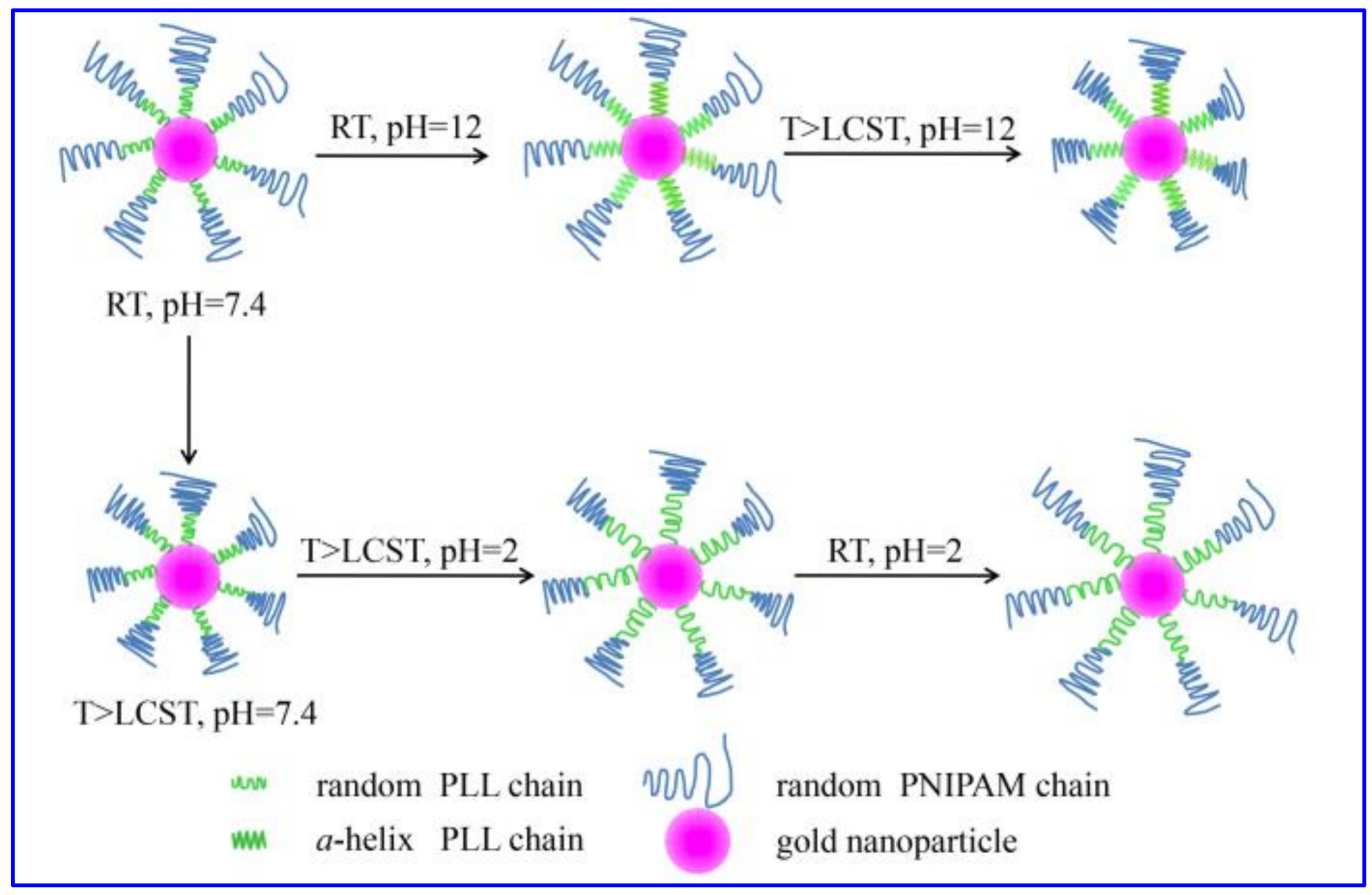
Illustration of pH- and temperature-responsive PNIPAM-PLL-AuNPs.

Gold nanoparticles protected by inner PLL block and outer PNIPAM block named as PNIPAM-PLL-AuNPs were successfully prepared and the dually pH- and temperature-responsive behaviors are illustrated as Scheme 1.

### 2.1. Preparation of PNIPAM-PLL-AuNPs

**Figure 1:**
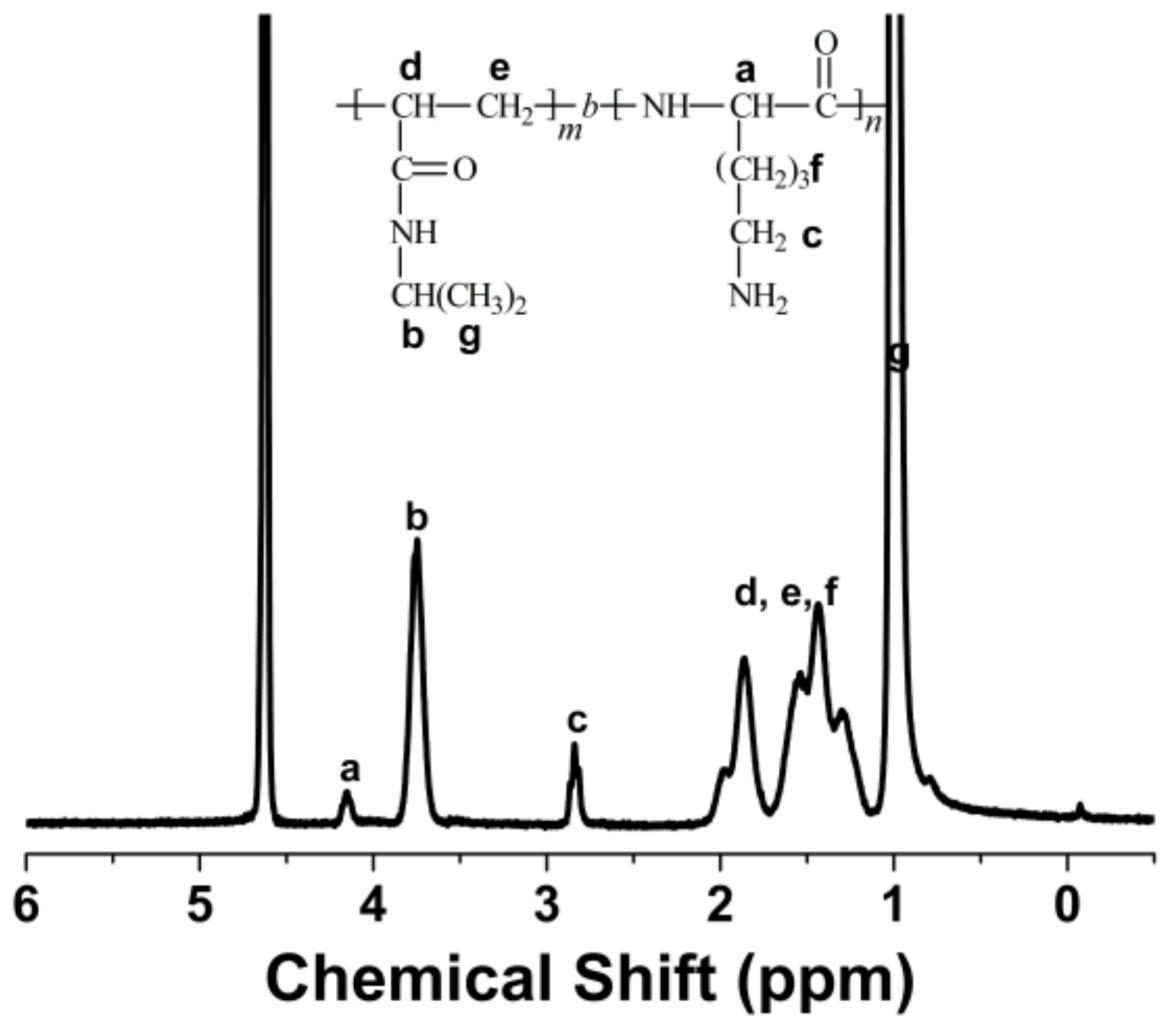
^1^H-NMR spectrum of PNIPAM_315_-*b*-PLL_25_ in D_2_O at 25 °C.

Firstly, PZLLys was prepared according to the method previously described [35]. The molecular weight and molecular weight distribution were characterized by GPC, resulting in *M*_n,GPC_ of 7000, and *M*_w_/*M*_n_, of 1.16. The average degree of polymerization of ZLLys was determined by ^1^H-NMR to be 25. Secondly, ATRP macro-initiator was successfully synthesized from reacting PZLLys with 2-chloropropionyl chloride. Subsequently, the ATRP of NIPAM was conducted to obtain the copolymer PNIPAM-*b*-PZLLys. The average degree of polymerization of NIPAM was determined by ^1^H-NMR to be 315. Molecular weight was characterized by GPC, resulting in *M*_n,GPC_ = 47,000 and *M*_w_/*M*_n_ = 1.14. Finally, PNIPAM-*b*-PLL was obtained after the de-protection with HBr/AcOH and TFA. Figure 1 shows ^1^H-NMR spectrum of PNIPAM-*b*-PLL in D_2_O. We can clearly see the characteristic signals of PNIPAM chains and PLL chains. The signals at 3.75 ppm (b) and 1.00 ppm (g) are ascribed to the protons of methine groups in the side chain of PNIPAM and the protons of methyl groups in the side chain of PNIPAM, respectively. Furthermore, the signal at 4.15 ppm is assigned to the protons of methine groups in the backbone from PLL and the signal at 2.84 ppm is attributed to the protons of methylene groups beside the primary amine from PLL, respectively. Based on the integration ratio of the signals of methine proton in PNIPAM (3.75 ppm, b) and the methylene protons in PLL (2.84 ppm, c), the average degree of polymerization of NIPAM was determined as 315.

PNIPAM-*b*-PLL protected gold nanoparticles were prepared by adding PNIPAM-*b*-PLL to the gold nanoparticle dispersion. Gold nanoparticles are stabilized through the interaction of lysine residues of PLL with the gold surface [36]. The resulting nanostructure consists of gold nanoparticle core, pH-responsive PLL inner layer and temperature-responsive PNIPAM outer corona, as shown in Scheme 1.

**Figure 2:**
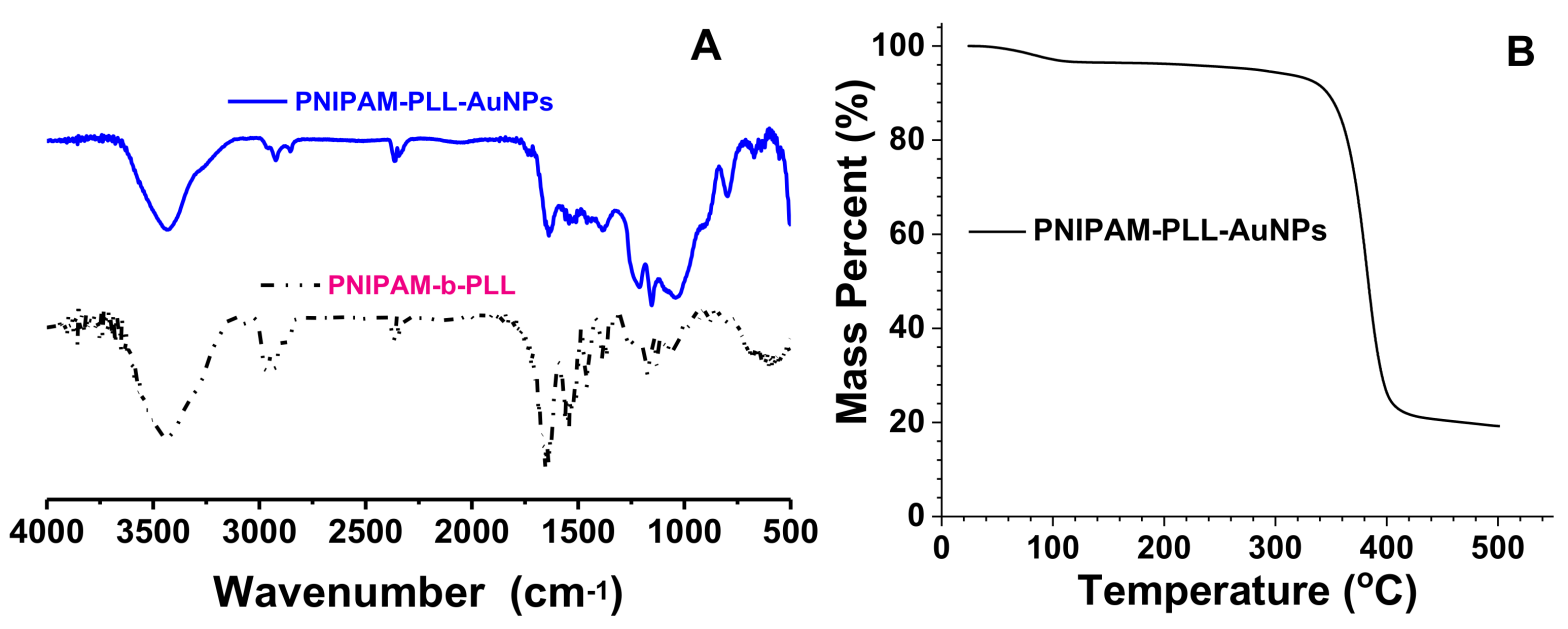
FT-IR spectra of PNIPAM-*b*-PLL and PNIPAM-PLL-AuNPs (**A**); TGA spectrum of PNIPAM-PLL-AuNPs (**B**).

The protection of gold nanoparticles with PNIPAM-*b*-PLL was primarily confirmed with FT-IR spectroscopy. After the gold nanoparticles were mixed with PNIPAM-*b*-PLL, they were centrifuged at 10,000 rpm for 30 min and the sedimented nanoparticles were dried at 50 °C overnight for FT-IR study. FT-IR spectroscopy shown in Figure 2A provides the reasonable evidence owning to the clearly elaborated characteristic absorbance of PNIPAM-*b*-PLL. The absorption peaks at 2973 and 2924 cm^−1^ are attributed to the symmetric stretching vibration of methyl and methylene. In addition, the weak absorption peak at 3280 cm^−1^ reveals the characteristic stretching vibration of amino group. The absorption peak at 1643 cm^−1^ and 1540 cm^−1^ are ascribed to the stretching vibration of carbonyl group and out-plane bending vibration of secondary amide, respectively. Besides, as we can see from TGA result in Figure 2B, the relative weight percent of grafted polymer is 80%. The weight loss during lower temperature range is due to the evaporation of physically adsorbed water and volatile matters. TGA diagram shows a rapid decrease of mass at 341 °C. As we know, the molecular weight of PNIPAM-*b*-PLL is 47,000. Besides, in present work, the averaged diameter of Au nanoparticle prepared from citrate is 18 nm as suggested by TEM observation. Combining TGA data with above information, the graft-density of PNIPAM-PLL-AuNPs was obtained as 2.97 polymer chain/nm^2^.

**Figure 3:**
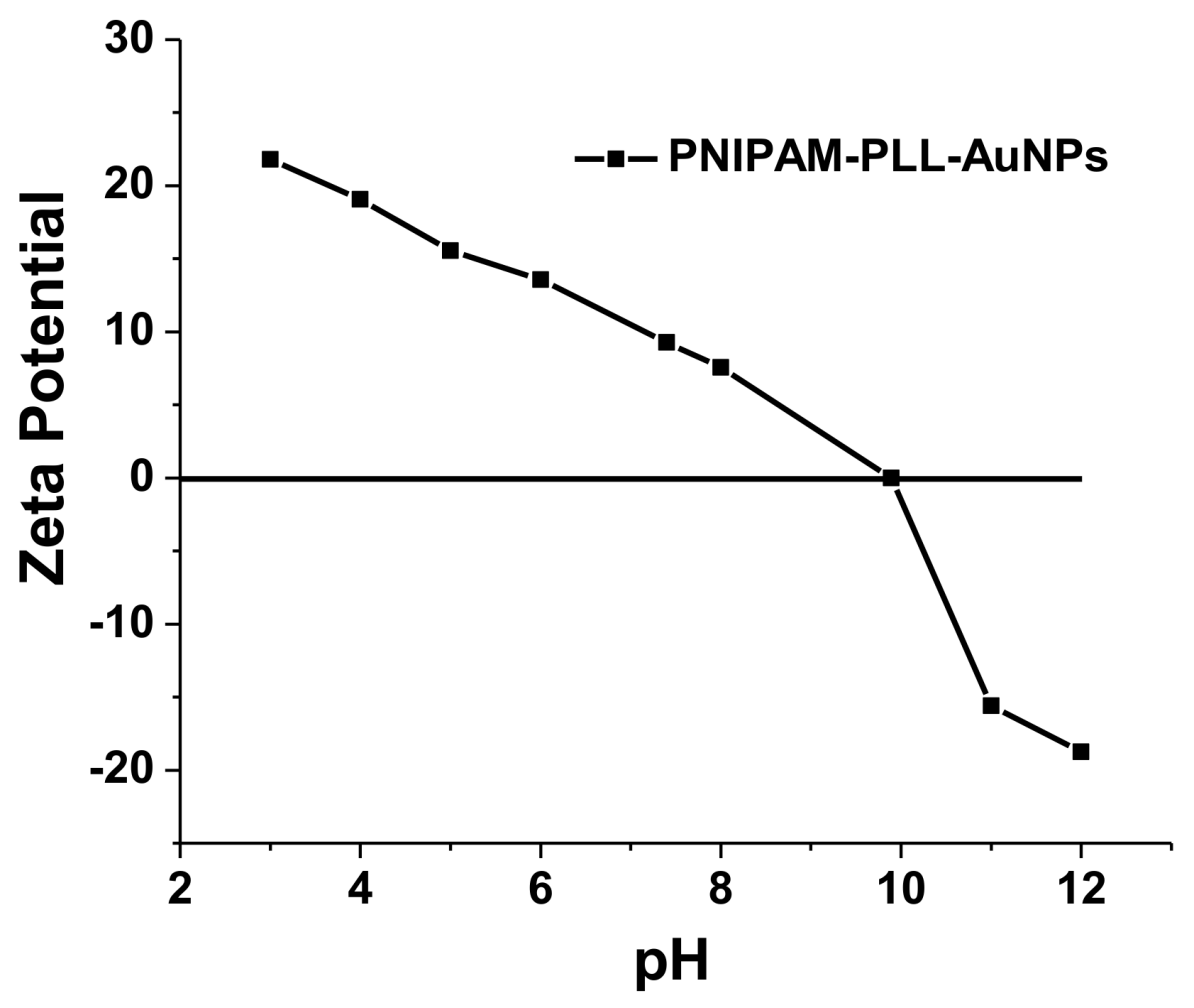
Curve of *Zeta*-potential versus pH for PNIPAM-PLL-AuNPs.

*Zeta* potential measurement was conducted to investigate the variation of the surface charge of PNIPAM-PLL-AuNPs with pH value. Since the amount of HCl or NaOH charged into neutral PNIPAM-PLL-AuNPs dispersions was very low, the influence of the change of ionic strength on the measurement could be ignored. As can be seen in Figure 3, the *Zeta* potential decreases with the increase of the pH value, owing to the deprotonation of the lysine resides and the gold dispersion exhibited an isoelectric point at pH = 9.89. Within the wide pH range from 2.0 to 12.0, PNIPAM-PLL-AuNPs showed good long-term colloidal stability at room temperature since the highly hydrated long PNIPAM chains on the gold surface provide the effective steric stabilization. The diblock copolymer protected gold nanoparticles remained colloidally stable even at its isoelectric point.

Combining the results of *Zeta* potential with FT-IR, it can be concluded that gold nanoparticles were successfully protected by PNIPAM-*b*-PLL copolymers. 

### 2.2. Comparison of the Stability of AuNPs Protected by PNIPAM-PLL Copolymer and PLL Homopolymer

**Figure 4:**
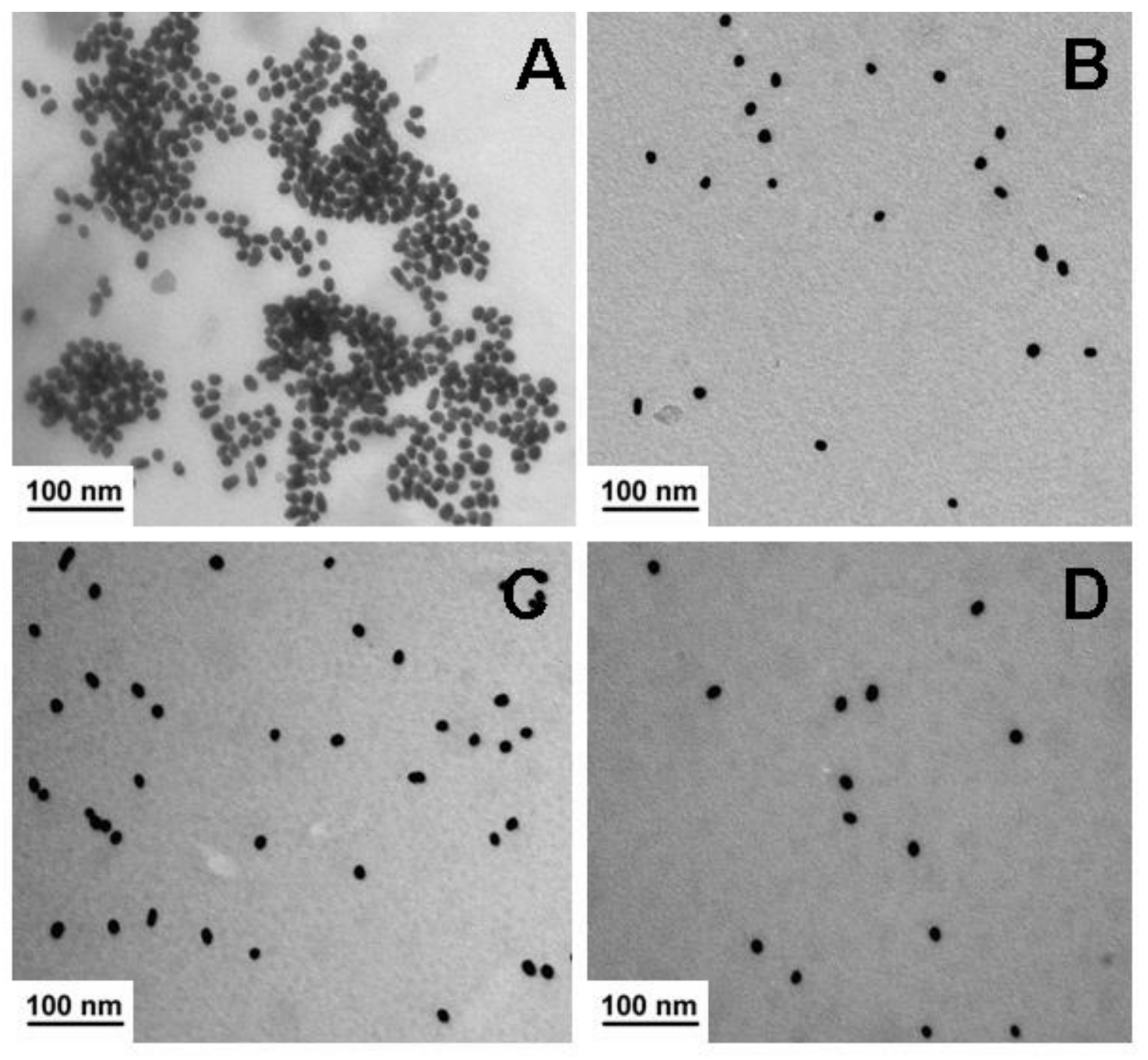
TEM images of gold nanoparticles protected by sodium citrate under pH = 7.0 (**A**) and PNIPAM-*b*-PLL chains under pH = 2.0 (**B**), pH = 7.4 (**C**) and pH = 12.0 (**D**).

Both the dispersions of citrate stabilized and PNIPAM-*b*-PLL stabilized gold nanoparticles were red in appearance. TEM observation was also employed to examine these gold nanoparticles. As shown in Figure 4A, citrate-capped nanoparticles show uniform size distribution with the averaged diameter of 18 nm, but they tend to aggregate into clusters under drying. On the contrary, PNIPAM-PLL-AuNPs show the less agglomeration at different pH values of 2, 7 and 12. It is clearly seen in Figure 4B–D, no obvious difference between the morphology and size at different pH values, which means this kind of polymer-protected AuNPs are extremely stable. Based on this property, it can serves as good catalyst for organic reaction such as reduction of 4-nitriphnol and degradation of dye [37,38].

**Figure 5:**
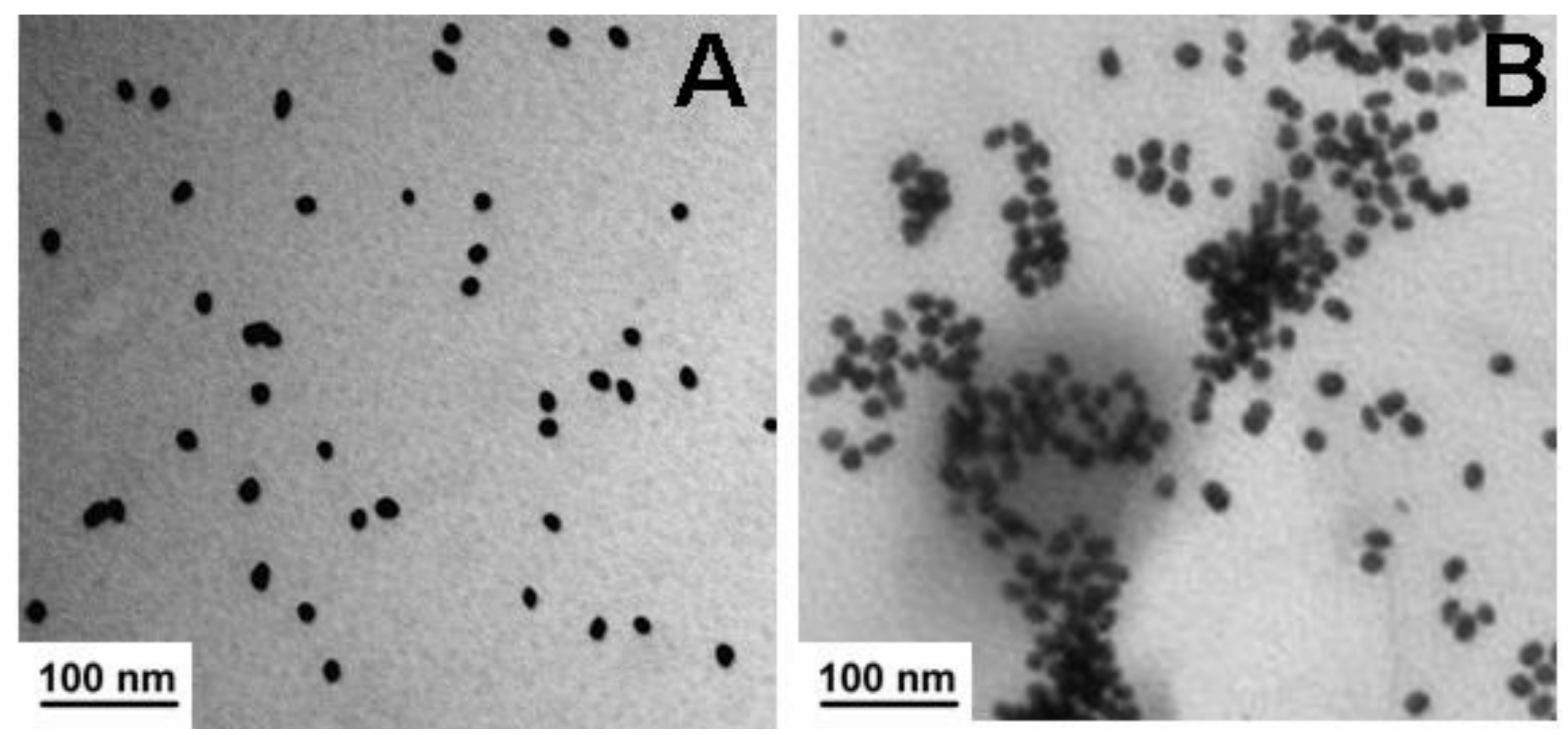
TEM images of PLL-AuNPs under pH = 2.0 (**A**) and pH = 12.0 (**B**).

As a comparison, Figure 5 shows TEM images of PLL stabilized AuNPs under acidic and alkaline conditions. At pH = 2.0, PLL-AuNPs were well-dispersed and no agglomeration was observed. However, PLL-AuNPs were found to undergo the aggregation at pH = 12.0.

### 2.3. Comparison of PLL Conformation between before and after Its Attachment onto AuNPs

PLL shows different conformations in aqueous solution under different pH values. Under acidic or neutral pH conditions, the repeating units of lysine get protonated, and the dominant conformation of PLL chains is random coil. Under alkaline conditions, the residues of PLL chains become deprotonated, resulting in the pH-induced coil-to-helix transitions [39,40,41]. The as-prepared gold nanoparticles obtained from the reduction of HAuCl_4_ with sodium citrate did not show any circular dichroism (CD) signal. The CD signals of PNIPAM-PLL-AuNPs should be attributed to the conformation change of PLL chains on the gold surface [42]. 

**Figure 6:**
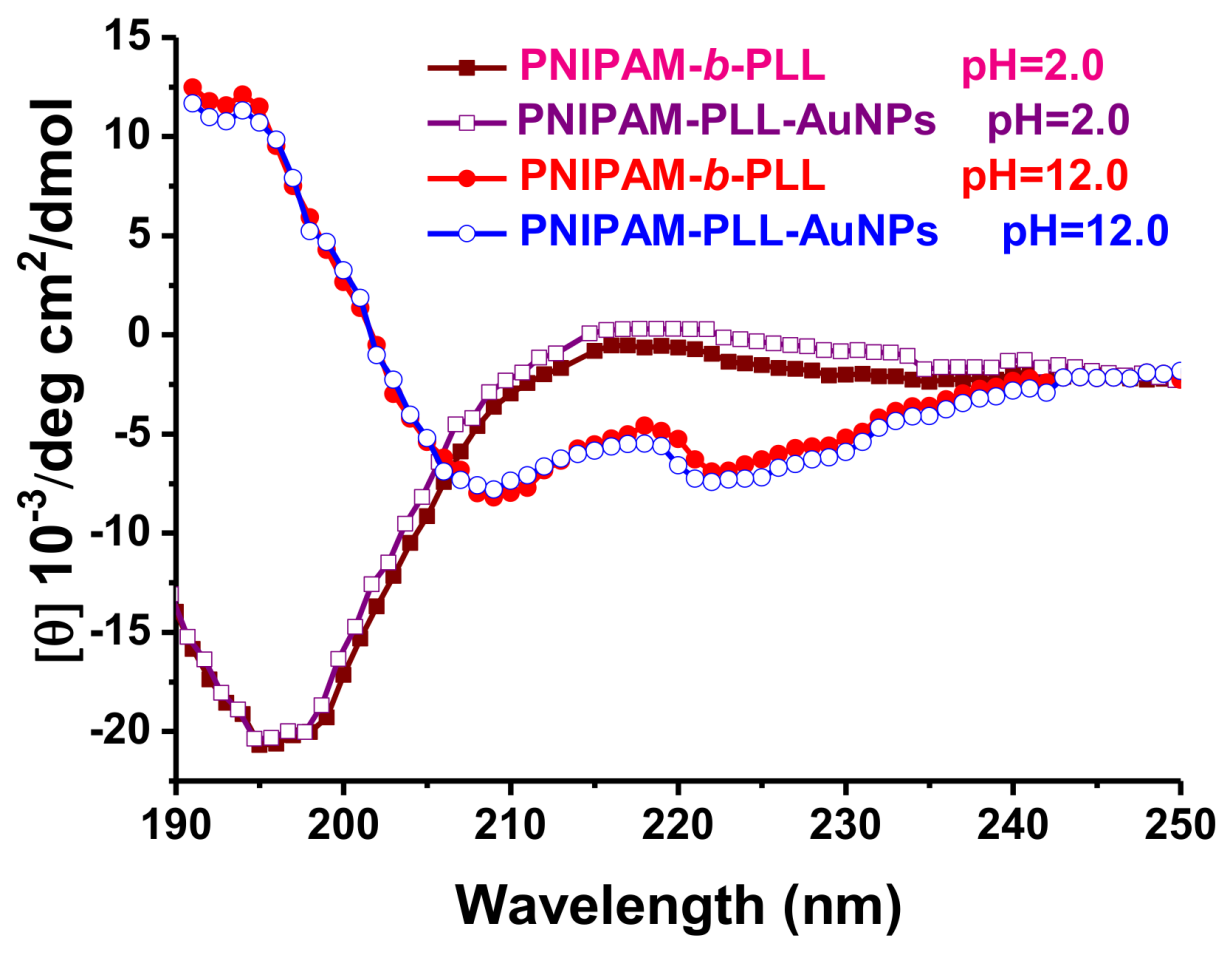
CD spectra of PNIPAM-*b*-PLL and PNIPAM-PLL-AuNPs under pH = 2.0 and pH = 12.0 at room temperature (Concentration: 2.0 mg/mL).

Figure 6 shows the CD spectra of PNIPAM-*b*-PLL block copolymer and PNIPAM-PLL-AuNPs at room temperature under different pH conditions. As shown in Figure 6, at pH = 2.0, CD spectra of PNIPAM-*b*-PLL block copolymer and PNIPAM-PLL-AuNPs both show a typical inflected curve with a positive maximum at 215 nm and a negative minimum at 196 nm, which can be assigned to the characteristic random coil conformation of polypeptide. On the contrary, at pH = 12.0, two negative minima at 208 and 220 nm are apparently detected, which can be attributed to the characteristic α-helix secondary structure of polypeptide. Pure PNIPAM-*b*-PLL and the block polymer attached to gold nanoparticles exhibit essentially the same CD spectra. This is to say that gold nanoparticles do not affect the secondary structure of PLL chains.

### 2.4. Dual pH- and Temperature-Response of PNIPAM-PLL-AuNPs Monitored with Optical Transmittance

**Figure 7:**
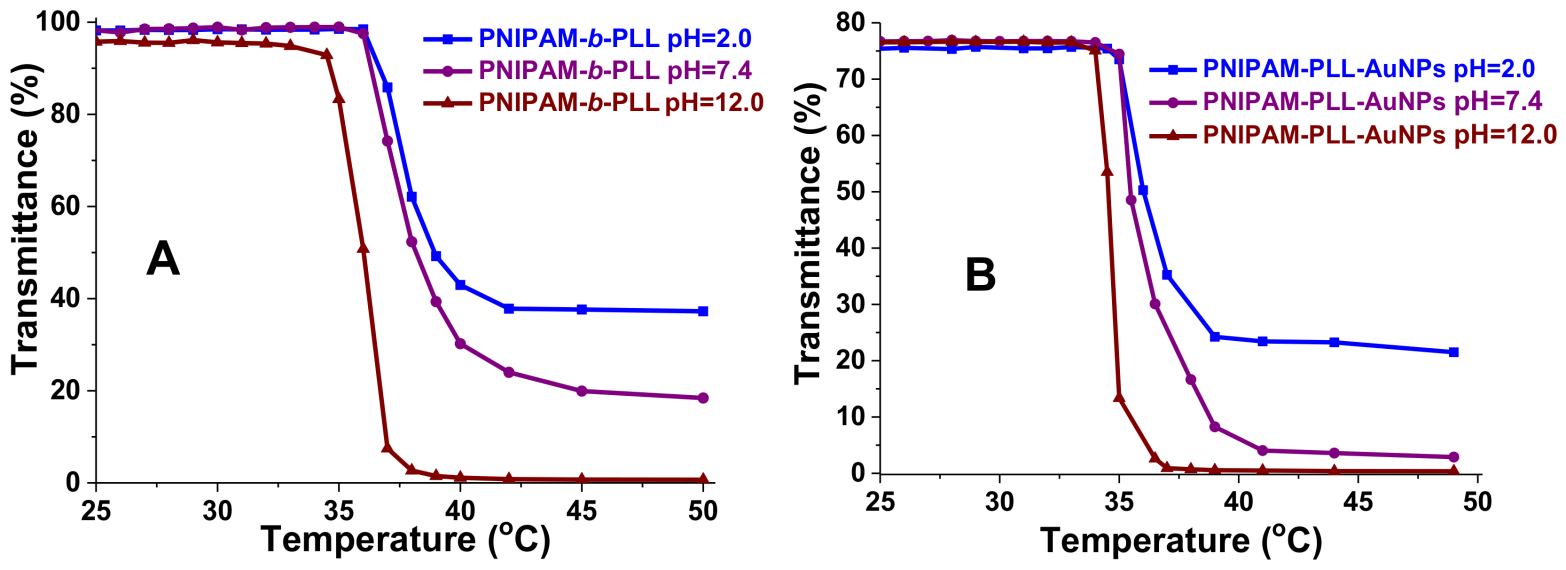
Temperature dependences of optical transmittance at 700 nm obtained for aqueous dispersions of PNIPAM-*b*-PLL (**A**) and PNIPAM-PLL-AuNPs (**B**) under different pH conditions at room temperature (Concentration: 2.0 mg/mL).

Diblock copolymer consisting of PNIPAM and PLL blocks was used to protect the gold nanoparticles. The conformation and solubility of PNIPAM in water can change with variation of temperature, while the solubility of the PLL block in water depends on the pH of the medium. It is reasonable to predict that the dispersion of PNIPAM-PLL-AuNPs should be temperature and pH-sensitive (Scheme 1). Generally, those properties can be determined by the transmittance change of aqueous solution. Transmittances of PNIPAM-*b*-PLL solution and PNIPAM-PLL-AuNPs dispersion at 700 nm were measured at three pH values and in the temperature range of 25–50 °C. As shown in Figure 7A, lower critical solution temperature (LCST) of PNIPAM-*b*-PLL is almost the same (37 °C) at pH = 2.0 and 7.0. At pH = 12.0, LCST value decreased to 35.5 °C. At lower pH, amine groups of PLL blocks get protonated and the polymer becomes more soluble in the aqueous medium. It is generally accepted that hydrophilic block content can result in a higher LCST value for PNIPAM chains [35,43].

Figure 7B shows the typical plots of the temperature-dependent optical transmittance of PNIPAM-PLL-AuNPs. Temperature sensitivity characteristics of PNIPAM-PLL-AuNPs under different pH conditions are different. LCST for thermosensitive gold nanoparticles under pH = 2.0 and 7.4 is 36 °C, while the transition temperature under pH = 12.0 is 34 °C. PNIPAM-PLL-AuNPs exhibit a relatively sharp decrease of transmittance under basic conditions and show more gradual decrease of transmittance under acidic or neutral conditions. This is similar to the results of Figure 7A. However, the LCST of PNIPAM-PLL-AuNPs is lower than that of the pure block polymer PNIPAM-*b*-PLL. When polymer chains bound to nanoparticles undertake the transition from an extended hydrophilic chain to a globular hydrophobic structure, they should collapse asymmetrically toward the nanoparticle surface [44,45,46]. In our experiment conditions, gold nanoparticle confines a cluster of polymers within a spherical space, which makes the hydrophilic to hydrophobic transition of PNIPAM chains easier.

### 2.5. Alteration of Surface Plasmon Resonance of PNIPAM-PLL-AuNPs with pH Value

**Figure 8:**
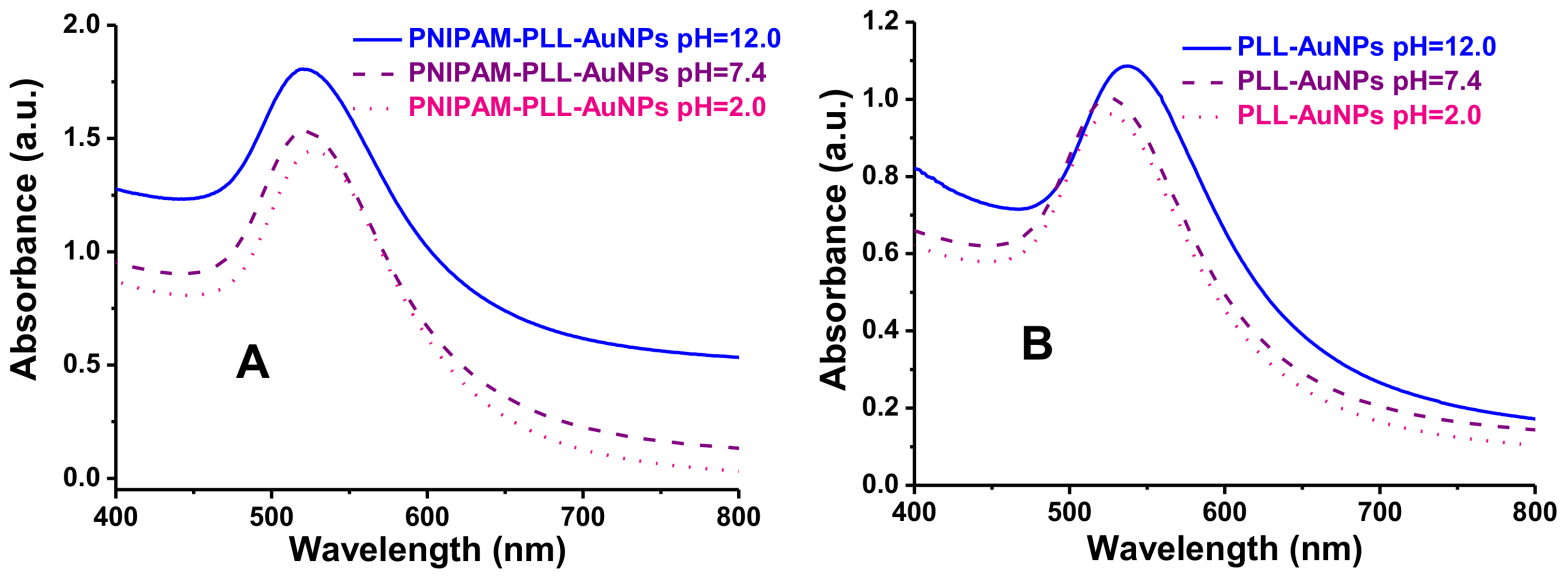
UV-vis absorption spectra as a function of pH of PNIPAM-PLL-AuNPs (**A**) and PLL-AuNPs (**B**) at room temperature (Concentration: 2.0 mg/mL).

The effect of pH on gold nanoparticles is monitored by optical spectroscopy. The pH is adjusted by adding HCl or NaOH to aqueous dispersions. Figure 8A compares typical UV-vis absorption spectra of PNIPAM-PLL-AuNPs at 25 °C under different pH values. The maximum wavelength (λ_max_) of the sample decreases as pH value increases, and the increase of pH of the solution is accompanied by a blue shift on the surface plasmon resonance. As the pH increases from 2 to 12, the λ_max_ shifts from 528 nm to 522 nm. It is reported that the surface plasmon absorption is an optical property for metallic nanoparticles due to an extensive electronic correlation and corresponds to a collective excitation of conduction electrons relative to the ionic core [2,47]. The dielectric constant of the solution can also affect the LSPR band of the gold nanoparticles [48,49]. Under acid conditions, PLL became protonated and hydrophilic, the distance between the nanoparticle is increased with the enhancement of electrostatic interaction which resulted in red shift of LSPR. Under alkaline conditions, PLL became hydrophobic, resulting in smaller number of water molecules around the gold nanoparticles [23]. Then, the shrinkage of the shell decreases the distance between individual nanoparticles, leading to the blue shift of the LSPR.

Compared with PNIPAM-PLL-AuNPs dispersions, under acidic or neutral conditions (pH = 2.0 and 7.4), no obvious change in the SPR band was observed in PLL-AuNPs dispersions (Figure 7B), indicating the PLL-AuNPs are well dispersed in the aqueous dispersion. Under this condition, pronated PLL also can act good stabilized agent for the nanoparticles, avoiding their aggregation. However, the maximum wavelength (λ_max_) of the sample increases as pH increases to 12, suggesting that AuNPs aggregated under this condition, which can be easily seen from Figure 5B. It is reported that when gold nanoparticles are too close to each other, the characteristic plasmon band shifts toward longer wavelengths because of an electronic dipole-dipole interaction between plasmons of neighboring gold nanoparticles. [50,51,52] The tendency of increasing aggregation suppressed the decreasing polarity of gold surrounding, resulting in red shift in LSPR. 

As can be seen from the above results, the maximum wavelength (λ_max_) of PNIPAM-PLL-AuNPs sample decreases as pH value increases, while for PLL-AuNPs, the maximum wavelength increases as pH increases. The results of UV-vis spectra are consistent with the TEM results. Moreover, it also demonstrated that PNIPAM is necessary for stabilized the gold nanoparticle at different pH values.

### 2.6. Alteration of Surface Plasmon Resonance of PNIPAM-PLL-AuNPs with Temperature

**Figure 9:**
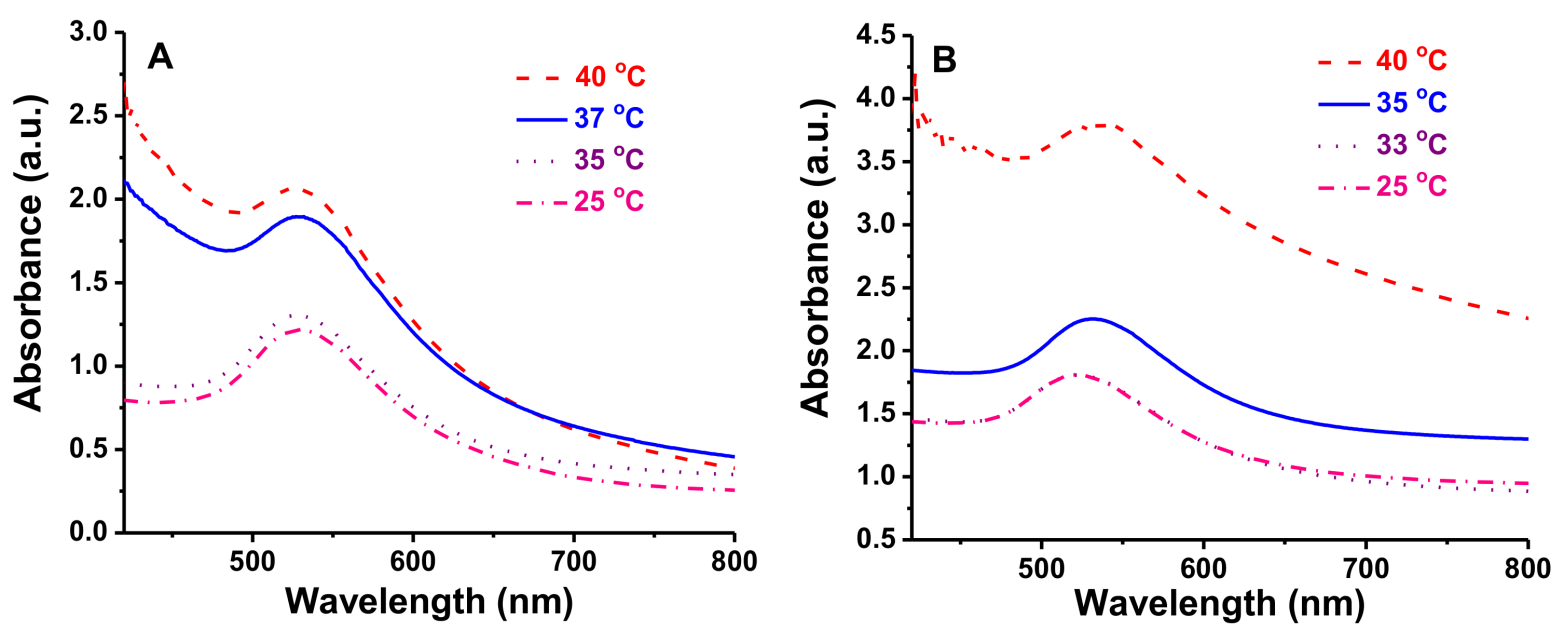
UV-vis absorption spectra of PNIPAM-PLL-AuNPs at various temperature at pH = 2.0 (**A**) and pH = 12.0 (**B**) (Concentration: 2.0 mg/mL).

To investigated if the thermal transition of PNIPAM affects the λ_max_ of LSPR band, absorption spectra of the PNIPAM-PLL-AuNPs dispersions were measured as a function of temperature at pH = 2.0 and 12.0. As can be seen from Figure 9A, at pH = 2.0, the hydrophilic PLL chains make the surroundings of gold core more hydrophilic, indicated by higher λ_max_ value at 25 °C. This is in accordance with the results of Figure 8A. Moreover, no significant shift of λ_max_ is detected at pH = 2.0 upon heating. Under acidic conditions (pH = 2.0), PLL is protonated at each temperature, the distance between polymer-protected AuNPs is remote due to electrostatic interaction. Although increasing the temperature, the collapse of PNIPAM chain would do little contribution to change the distance, the λ_max_ is approximately equal. On the other hand, at pH = 12.0, a clear blue shift was observed at 25 °C due to the hydrophobic PLL chains. Upon heating, the shift of λ_max_ to higher wavelengths was observed. When altering the condition to alkaline (pH = 12), there no protonation happens, so the distance from charge repulsion is nonexistent. Once increasing the temperature, PNIPAM chains collapse, while the distance between polymer-protected Au nanoparticle decrease, the blue shift of LSPR happens. 

**Figure 10:**
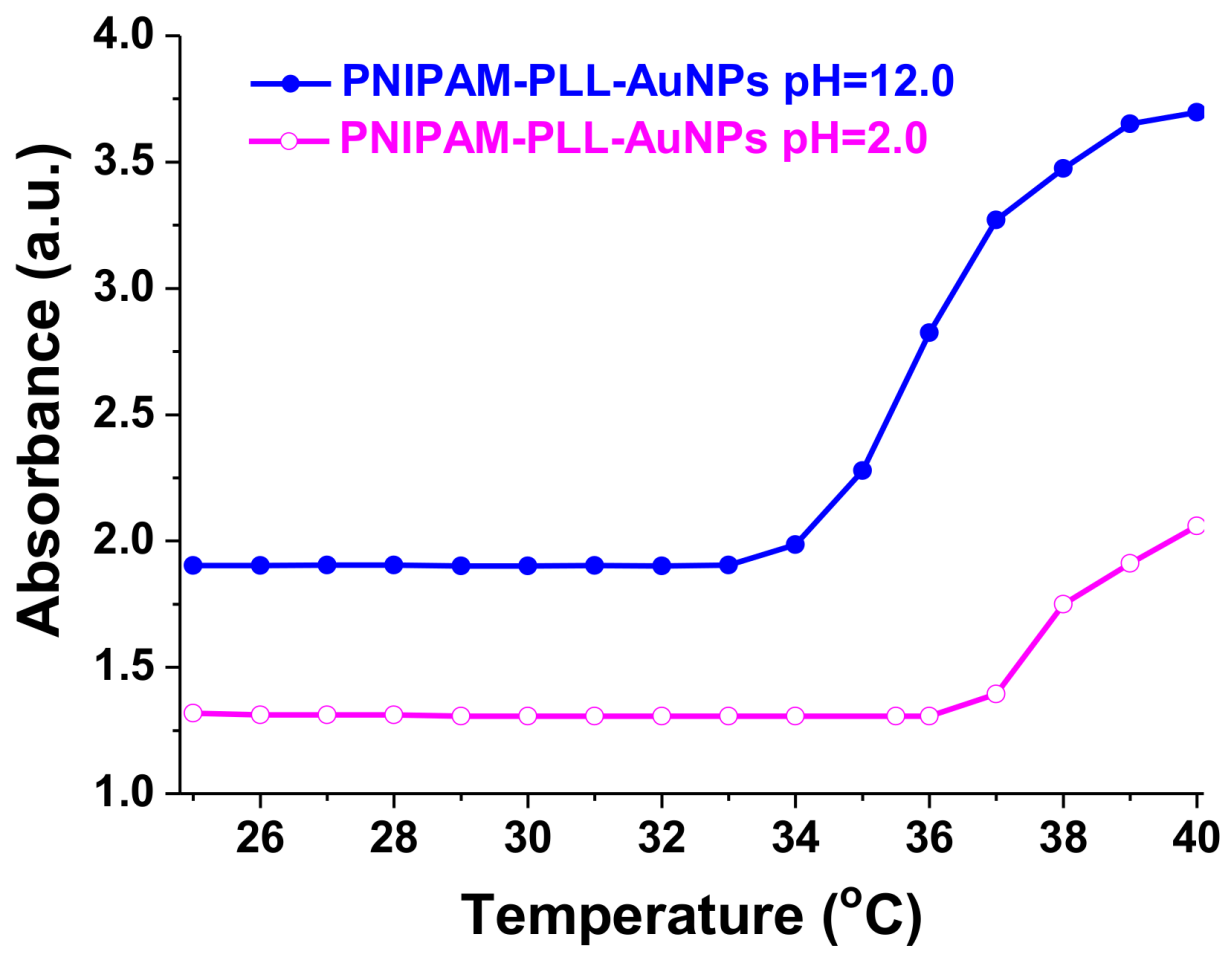
Variation of absorbance PNIPAM-PLL-AuNPs at λ_max_ as a function of temperature under two pH conditions (pH = 2.0 and 12.0) (Concentration: 2.0 mg/mL).

To display the correlation between the absorbance of the gold dispersions at λ_max_ and temperature and pH, the plots of the absorbance at λ_max_ versus temperature at pH = 2.0 and 12.0 was shown in Figure 10. Absorbance of the sample under both conditions increases with the increase of temperature, as expected. The onset temperature of the absorbance increment under pH = 2.0 and 12.0 is 35.5 °C and 34 °C, respectively. The multi-responsive sensor system has been established. 

**Figure 11:**
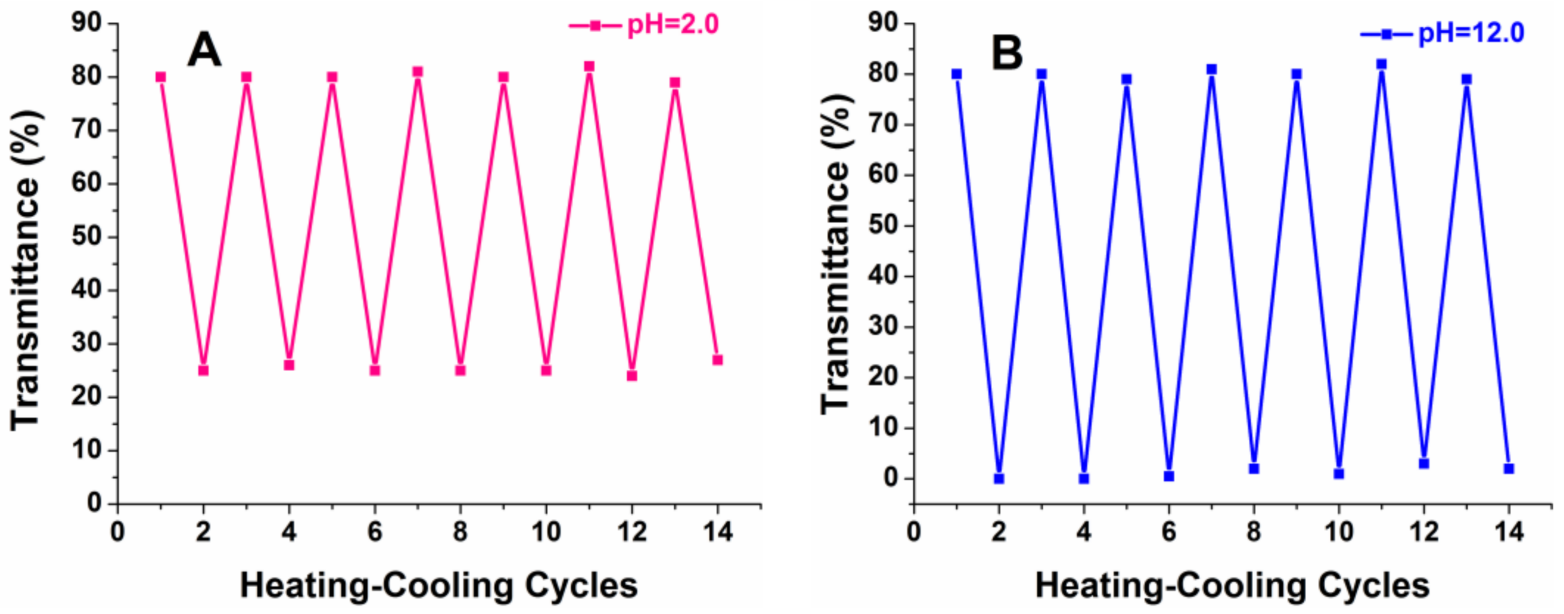
Transmittance change during the heating-cooling cycles of PNIPAM-PLL-AuNPs between 25 °C and 50 °C under pH = 2.0 (**A**) and pH = 12.0 (**B**). (Concentration: 2.0 mg/mL).

Moreover, the AuNPs dispersions are stable enough for application. The opaque suspension of PNIPAM-PLL-AuNPs does not precipitate, and this temperature-dependent clear-opaque transition of PNIPAM-PLL-AuNPs is completely reversible. Figure 11 shows thermoresponsive changes of the absorption maximum of PNIPAM-PLL-AuNPs dispersions at pH = 2.0 and pH = 12.0. In the transition regime, the optical transparency is a function of temperature. The temperature change cycle is observed between 25 and 50 °C. Under pH = 2.0, the optical transparency changes reversibly between ~80% and ~25% transmittance, while under pH = 12.0, the optical transparency changes reversibly between ~80% and ~0% transmittance. The reversible thermo-responsive property of PNIPAM-PLL-AuNPs is owing to the change of the conformation of PNIPAM chain induced by the stimuli of temperature. At 25 °C, the highly hydrated long PNIPAM chains extend in the solution, effectively stabilize the gold nanoparticles, on the other hand, at 50 °C, PNIPAM chains collapse, weakening the protection of gold nanoparticles.

## 3. Materials and Methods

### 3.1. Materials

Poly(ε-benzyloxycarbonyl-l-lysine) (PZLLys) were prepared according to the method previously described (*M*_n_ = 7000, and *M*_w_/*M*_n_ = 1.16) [35]. *N*-Isopropylacrylamide (NIPAM, 97%, Kohjin Co., Kumamoto, Japan) was purified by recrystallization from a benzene/*n*-hexane mixture (65/35 *v*/*v*). 2-Chloropropionyl chloride (98%) was purchased from Sigma-Aldrich (Shanghai, China) and used as received. *N*,*N*′-Dimethylformamide (DMF) was dried and distilled from anhydrous magnesium sulfate under reduced pressure. Hydrobromic acid in glacial acetic acid (HBr/AcOH, 45% *w*/*v*) and trifluoroacetic acid (TFA) was purchased from Alfa Aesar (Shanghai, China) and used without further purification. Sodium citrate tribasic dihydrate and hydrogen tetrachloroaurate trihydrate were obtained from Shanghai Chemical Reagent Company (Shanghai, China). The ligand tris[2-(dimethylamino)ethyl]amine (Me_6_TREN) was synthesized as reported in the literature [53]. All the other reagents were of analytical grade and used as received.

### 3.2. Synthesis of PNIPAM-b-PLL Copolymer

PZLLys was reacted with 2-chloropropionyl chloride to provide the atom transfer radical polymerization (ATRP) initiator of PZLLys-Cl according to the literature [35]. PNIPAM-*b*-PZLLys was synthesized as follows. NIPAM (1.89 g, 2.39 × 10^−2^ mol), ATRP initiator of PZLLys-Cl (0.3 g, 3.99 × 10^−5^ mol), Me_6_TREN (18.5 mg, 7.98 × 10^−5^ mol), CuCl (7.9 mg, 7.98 × 10^−5^ mol) and DMF (4 mL) were added into a 10 mL polymerization tube. After three freeze-vacuum-thaw cycles, the tube was sealed under vacuum and then immersed in a water bath thermostatted at 25 °C. After 24 h, the tube was opened, and the reaction mixture was diluted with THF, passed through a neutral alumina column to remove copper salts. After most of THF was removed by rotary evaporation, the polymer was obtained by precipitation into ethyl ether and dried under vacuum at 25 °C for 24 h. The removal of the protecting ε-(benzyloxycarbonyl) groups of PZLLys was conducted with HBr/AcOH and TFA according to the literature [25].

### 3.3. Synthesis and Functionalization of Gold Nanoparticles

Gold nanoparticles were prepared via the common technique of citrate reduction according to the literature [54]. In a 100 mL round-bottom flask equipped with a condenser, 48 mL of 1 mM HAuCl_4_ was brought to a rolling boil with vigorous stirring. Rapid addition of 4.8 mL of 38.8 mM sodium citrate to the vortex of the solution resulted in a rapid color change from pale yellow to wine-red. After addition, boiling was continued for 10 min, then the stirring was continued for an additional 1 h. When the solution reached room temperature, it was filtered through a 0.8 μm Millipore membrane filter (Millex-AA, Merck Millipore, Billerica, MA, USA). The obtained gold nanoparticles were stored at room temperature in a dark bottle. The maximum optical absorbance of gold nanoparticles was observed at 520 nm and the diameter was about 18 nm as determined by UV-vis spectroscope and TEM analysis, respectively.

For polymer functionalization, 75 μL of PNIPAM-*b*-PLL (80 mg/mL) was added rapidly into 1.5 mL gold dispersion. The molar ratio of polymer/gold was kept at 1:10. The solution was stirred violently at dark place for two days. The reaction solution was purified by centrifuged at 10,000 rpm for 1 h at room temperature and the supernatant was removed. Free polymer chains which were not conjugated to gold nanoparticles were removed by this process. This process was repeated two times. The final product named as PNIPAM-PLL-AuNPs was kept at 4 °C in the dark. PLL-AuNPs were prepared in the same way.

### 3.4. Characterization

Fourier transform infrared (FT-IR) spectra were recorded on a Bruker VECTOR-22 IR spectrometer (Bruker, Karlsruhe, Germany). Proton nuclear magnetic resonance (^1^H-NMR) spectra were recorded at 25 °C on a Bruker AV300 NMR spectrometer (Bruker, Karlsruhe, Germany) (resonance frequency of 300 MHz). The molecular weight distribution, relative number- and weight-average molecular weights (*M*_n_, and *M*_w_), were determined by gel permeation chromatography instrument (GPC, Waters 1515, Waters, Milford, MA, USA) equipped with a refractive index detector (RI, Wyatt WREX-02, Wyatt, Santa Barbara, CA, USA). DMF was used as the eluent at a flow rate of 1.0 mL min^−1^ and the calibration was carried out using linear polystyrene standards. Circular dichroism (CD) spectra were recorded at 25 °C with a Jasco J-720 spectrophotometer (Jasco, Tokyo, Japan). The UV-vis spectra and transmittance were acquired on a Unico UV/vis 2802PCS spectrophotometer (Unico, Dayton, NJ, USA). The optical transmittance of the aqueous solutions of the obtained polymer at a wavelength of 700 nm was obtained using a thermostatically controlled cuvette and the heating rate was 0.2 °C/min. *Zeta* potential of AuNPs were measured on a Malvern Zetasizer NanoZS instrument (Malvern Panalytical Ltd., Malvern, United Kingdom). The pH value of AuNPs dispersion was adjusted by the addition of 1 M HCl or 1 M NaOH aqueous solution. The morphology of the obtained AuNPs was observed on JEOL 2010 Transmission Electron Microscopy (TEM) instrument (JEOL, Tokyo, Japan). The samples for TEM observations were prepared by placing 10 μL of AuNPs dispersion on copper grids coated with carbon film. No staining was applied. An accelerating voltage of 200 kV was applied during TEM observation. Thermal gravimetric analyses (TGA) was performed on a diamond TG/DTA (Perkin-Elmer, Waltham, MA, USA) with heating rate of 10 °C/min under N_2_ atmosphere.

## 4. Conclusions

Gold nanoparticles coated with dually pH- and temperature-responsive PNIPAM-*b*-PLL copolymer were prepared by bounding the residues of PLL onto the gold nanoparticle surface. PNIPAM block provides the hybrid gold nanoparticle with temperature response while PLL block entitles the hybrid gold nanoparticle pH response owning to the coil-to-helix conformation transition. The pH response was confirmed via CD spectra by adjusting the pH of the hybrid gold nanoparticle dispersion between pH values of 2 and 12 at room temperature. TEM and UV-vis absorption spectra verified the good stability of these hybrid gold nanoparticles under different pH values. Good dispersion ability as well as the decrease of λ_max_ with increment of pH value of these hybrid gold nanoparticles confirmed the high stability even at strong alkaline (pH = 12) conditions, in virtue of the highly hydrophilic long PNIPAM chains on the gold surface. Moreover, temperature response was monitored by transmittance studies at fixed pH under different temperatures. The results affirm the good stability as well as the reversible temperature-dependent clear-opaque transition of the hybrid gold nanoparticles. All the above-mentioned characteristics entitle these hybrid gold nanoparticles to potential use for stimuli-responsive applications. 

## References

[1] Hayat M.A. (1989). Colloid Gold: Principles, Methods, and Applications.

[2] Kreibig U., Vollmer M. (1995). Optical Properties of Metal Clusters.

[3] Shipway A.N., Katz E., Willner I. (2000). Nanoparticle arrays on surfaces for electronic, optical, and sensor applications. ChemPhysChem.

[4] Adams D.M., Brus L., Chidsey C.E.D., Creager S., Creutz C., Kagan C.R., Kamat P.V., Lieberman M., Lindsay S., Marcus R.A. (2003). Charge transfer on the nanoscale: Current status. J. Phys. Chem. B.

[5] Daniel M.C., Astruc D. (2004). Gold nanoparticles: Assembly, supramolecular chemistry, quantum-size-related properties, and applications toward biology, catalysis, and nanotechnology. Chem. Rev..

[6] Corti C.W., Holliday R.J. (2004). Commercial aspects of gold applications: From materials science to chemical science. Gold Bull..

[7] Feldheim D.L., Keating C.D. (1998). Self-assembly of single electron transistors and related devices. Chem. Soc. Rev..

[8] Maier S.A., Brongersma M.L., Kik P.G., Meltzer S., Requicha A.A.G., Atwater H.A. (2001). Plasmonics—A route to nanoscale optical devices. Adv. Mater..

[9] Kesharwani P., Jain K., Jain N.K. (2014). Dendrimer as nanocarrier for drug delivery. Prog. Polym. Sci..

[10] Marcelo G., Munoz-Bonilla A., Fernandez-Garcia M. (2012). Magnetite-Polypeptide Hybrid Materials Decorated with Gold Nanoparticles: Study of Their Catalytic Activity in 4-Nitrophenol Reduction. J. Phys. Chem. C.

[11] Brust M., Walker M., Bethell D., Schiffrin D.J., Whyman R. (1994). Synthesis of Thiol-Derivatized Gold Nanoparticles in a 2-Phase Liquid-Liquid System. J. Chem. Soc. Chem. Commun..

[12] Templeton A.C., Wuelfing M.P., Murray R.W. (2000). Monolayer protected cluster molecules. Acc. Chem. Res..

[13] Zhou Y., Itoh H., Uemura T., Naka K., Chujo Y. (2002). Synthesis of novel stable nanometer-sized metal (M = Pd, Au, Pt) colloids protected by a π-conjugated polymer. Langmuir.

[14] Mossmer S., Spatz J.P., Moller M., Aberle T., Schmidt J., Burchard W. (2000). Solution behavior of poly(styrene)-*block*-poly(2-vinylpyridine) micelles containing gold nanoparticles. Macromolecules.

[15] Miyazaki A., Nakano Y. (2000). Morphology of platinum nanoparticles protected by poly(*N*-isopropylacrylamide). Langmuir.

[16] De la Fuente J.M., Barrientos A.G., Rojas T.C., Rojo J., Canada J., Fernandez A., Penades S. (2001). Gold glyconanoparticles as water-soluble polyvalent models to study carbohydrate interactions. Angew. Chem. Int. Ed..

[17] Cao Y.W., Jin R., Mirkin C.A. (2001). DNA-modified core-shell Ag/Au nanoparticles. J. Am. Chem. Soc..

[18] Bartz M., Kuther J., Nelles G., Weber N., Seshadri R., Tremel W. (1999). Monothiols derived from glycols as agents for stabilizing gold colloids in water: Synthesis, self-assembly and use as crystallization templates. J. Mater. Chem..

[19] Liu J., Ong W., Roman E., Lynn M.J., Kaifer A.E. (2000). Cyclodextrin-modified gold nanospheres. Langmuir.

[20] Thomas K.G., Kamat P.V. (2000). Making gold nanoparticles glow: Enhanced emission from a surface-bound fluoroprobe. J. Am. Chem. Soc..

[21] Hostetler M.J., Murray R.W. (1997). Colloids and self-assembled monolayers. Curr. Opin. Colloid Interface Sci..

[22] Hostetler M.J., Green S.J., Stokes J.J., Murray R.W. (1996). Monolayers in three dimensions: Synthesis and electrochemistry of ω-functionalized alkanethiolate-stabilized gold cluster compounds. J. Am. Chem. Soc..

[23] Nuopponen M., Tenhu H. (2007). Gold nanoparticles protected with pH and temperature-sensitive diblock copolymers. Langmuir.

[24] Li L.Y., He W.D., Li W.T., Zhang K.R., Pan T.T., Ding Z.L., Zhang B.Y. (2010). Thermal and pH-sensitive gold nanoparticles from H-shaped block copolymers of (PNIPAM/PDMAEMA)-*b*-PEG-*b*-(PNIPAM/PDMAEMA). J. Polym. Sci. Part A Polym. Chem..

[25] Song L.C., Sun H., Chen X.L., Han X., Liu H.L. (2015). From multi-responsive tri- and diblock copolymers to diblock-copolymer-decorated gold nanoparticles: The effect of architecture on micellization behaviors in aqueous solutions. Soft Matter.

[26] Kuila A., Maity N., Chatterjee D.P., Nandi A.K. (2017). pH and Temperature Responsiveness in AgNPs Stabilized by a New Poly(Vinylidene Fluoride) Random Graft Copolymer. J. Polym. Sci. Pol. Chem..

[27] Kitayama Y., Takeuchi T. (2014). Localized Surface Plasmon Resonance Nanosensing of C-Reactive Protein with Poly(2-methacryloyloxyethyl phosphorylcholine)-Grafted Gold Nanoparticles Prepared by Surface-Initiated Atom Transfer Radical Polymerization. Anal. Chem..

[28] Wang Z.Z., Chen Z.W., Liu Z., Shi P., Dong K., Ju E.G., Ren J.S., Qu X.G. (2014). A multi-stimuli responsive gold nanocage-hyaluronic platform for targeted photothermal and chemotherapy. Biomaterials.

[29] Aioub M., El-Sayed M.A. (2016). A Real-Time Surface Enhanced Raman Spectroscopy Study of Plasmonic Photothermal Cell Death Using Targeted Gold Nanoparticles. J. Am. Chem. Soc..

[30] Davidson B., Fasman G.D. (1967). Conformational Transitions of Uncharged Poly-l-Lysine. α Helix-Random Coil-β Structure. Biochemistry.

[31] Greenfie N., Fasman G.D. (1969). Computed Circular Dichroism Spectra for Evaluation of Protein Conformation. Biochemistry.

[32] Guo Y., Xia F., Xu L., Li J., Yang W.S., Jiang L. (2010). Switchable Wettability on Cooperative Dual-Responsive Poly-l-lysine Surface. Langmuir.

[33] Chen L.L., Chen T., Fang W.X., Wen Y., Lin S.L., Lin J.P., Cai C.H. (2013). Synthesis and pH-Responsive “Schizophrenic” Aggregation of a Linear-Dendron-Like Polyampholyte Based on Oppositely Charged Polypeptides. Biomacromolecules.

[34] Malinsky M.D., Kelly K.L., Schatz G.C., Van Duyne R.P. (2001). Chain length dependence and sensing capabilities of the localized surface plasmon resonance of silver nanoparticles chemically modified with alkanethiol self-assembled monolayers. J. Am. Chem. Soc..

[35] Li L.Y., He W.D., Li J.A., Zhang B.Y., Pan T.T., Sun X.L., Ding Z.L. (2010). Shell-Cross-Linked Micelles from PNIPAM-*b*-(PLL)(2) Y-Shaped Miktoarm Star Copolymer as Drug Carriers. Biomacromolecules.

[36] Xu L., Guo Y., Xie R.G., Zhuang J.Q., Yang W.S., Li T.J. (2002). Three-dimensional assembly of Au nanoparticles using dipeptides. Nanotechnology.

[37] Shen W.L., Qu Y.Y., Pei X.F., Li S.Z., You S.N., Wang J.W., Zhang Z.J., Zhou J.T. (2017). Catalytic reduction of 4-nitrophenol using gold nanoparticles biosynthesized by cell-free extracts of *Aspergillus* sp. WL-Au. J. Hazard Mater..

[38] Dutt S., Siril P.F., Sharma V., Periasamy S. (2015). Gold(core)-polyaniline(shell) composite nanowires as a substrate for surface enhanced Raman scattering and catalyst for dye reduction. New J. Chem..

[39] Smirnovas V., Winter R., Funck T., Dzwolak W. (2005). Thermodynamic properties underlying the α-helix-to-β-sheet transition, aggregation, and amyloidogenesis of polylysine as probed by calorimetry, densimetry, and ultrasound velocimetry. J. Phys. Chem. B.

[40] Dzwolak W., Muraki T., Kato M., Taniguchi Y. (2004). Chain-length dependence of α-helix to β-sheet transition in polylysine: Model of protein aggregation studied by temperature-tuned FTIR spectroscopy. Biopolymers.

[41] Rao J.Y., Zhang Y.F., Zhang J.Y., Liu S.Y. (2008). Facile Preparation of Well-Defined AB(2) Y-Shaped Miktoarm Star Polypeptide Copolymer via the Combination of Ring-Opening Polymerization and Click Chemistry. Biomacromolecules.

[42] Li T.H., Park H.G., Lee H.S., Choi S.H. (2004). Circular dichroism study of chiral biomolecules conjugated nanoparticles. Nanotechnology.

[43] Xia Y., Burke N.A.D., Stover H.D.H. (2006). End group effect on the thermal response of narrow-disperse poly(*N*-isopropylacrylamide) prepared by atom transfer radical polymerization. Macromolecules.

[44] Wang W., Wan W., Zhou H.H., Niu S.Q., Li A.D.Q. (2003). Alternating DNA and π-conjugated sequences. Thermophilic foldable polymers. J. Am. Chem. Soc..

[45] Wang W., Han J.J., Wang L.Q., Li L.S., Shaw W.J., Li A.D.Q. (2003). Dynamic π-π stacked molecular assemblies emit from green to red colors. Nano Lett..

[46] Wang W., Li L.S., Helms G., Zhou H.H., Li A.D.Q. (2003). To fold or to assemble?. J. Am. Chem. Soc..

[47] Liz-Marzan L.M. (2006). Tailoring surface plasmons through the morphology and assembly of metal nanoparticles. Langmuir.

[48] Templeton A.C., Pietron J.J., Murray R.W., Mulvaney P. (2000). Solvent refractive index and core charge influences on the surface plasmon absorbance of alkanethiolate monolayer-protected gold clusters. J. Phys. Chem. B.

[49] Ghosh S.K., Nath S., Kundu S., Esumi K., Pal T. (2004). Solvent and ligand effects on the localized surface plasmon resonance (LSPR) of gold colloids. J. Phys. Chem. B.

[50] Heath J.R., Knobler C.M., Leff D.V. (1997). Pressure/temperature phase diagrams and superlattices of organically functionalized metal nanocrystal monolayers: The influence of particle size, size distribution, and surface passivant. J. Phys. Chem. B.

[51] Collier C.P., Saykally R.J., Shiang J.J., Henrichs S.E., Heath J.R. (1997). Reversible tuning of silver quantum dot monolayers through the metal-insulator transition. Science.

[52] Yusa S.I., Fukuda K., Yamamoto T., Iwasaki Y., Watanabe A., Akiyoshi K., Morishima Y. (2007). Salt effect on the heat-induced association Behavior of gold nanoparticles coated with Poly(*N*-isopropylacrylamide) prepared via reversible addition—Fragmentation chain transfer (RAFT) radical polymerization. Langmuir.

[53] Ciampoli M., Nardi N. (1966). 5-Coordinated High-Spin Complexes of Bivalent Cobalt Nickel and Copper with Tris(2-Dimethylaminoethyl)Amine. Inorg. Chem..

[54] Grabar K.C., Freeman R.G., Hommer M.B., Natan M.J. (1995). Preparation and Characterization of Au Colloid Monolayers. Anal. Chem..

